# *catena*-Poly[[(ethanol-κ*O*)[3-(1-phenyl-1*H*-pyrazol-3-yl)benzoic acid-κ*O*]lithium]-μ-3-(1-phenyl-1*H*-pyrazol-3-yl)benzoato-κ^2^ *O*:*O*′]

**DOI:** 10.1107/S1600536809026385

**Published:** 2009-07-15

**Authors:** Hoong-Kun Fun, Kasthuri Balasubramani, Sankappa Rai, Prakash Shetty, Arun M. Isloor

**Affiliations:** a X-ray Crystallography Unit, School of Physics, Universiti Sains Malaysia, 11800 Universiti Sains Malaysia, Penang, Malaysia; b Syngene International Ltd, Biocon Park, Plot Nos. 2 & 3, Bommasandra 4th Phase, Jigani Link Rd, Bangalore 560 100, India; c Department of Printing, Manipal Institute of Technology, Manipal 576 104, India; d Department of Chemistry, National Institute of Technology–Karnataka, Surathkal, Mangalore 575 025, India

## Abstract

The asymmetric unit of the title polymeric compound, [Li_2_(C_16_H_11_N_2_O_2_)_2_(C_16_H_12_N_2_O_2_)_2_(CH_3_CH_2_OH)_2_]_*n*_, contains two Li^I^ ions, two 3-(1-phenyl-1*H*-pyrazol-3-yl)benzoate ions, two 3-(1-phenyl-1*H*-pyrazol-3-yl)benzoic acid mol­ecules and two ethanol mol­ecules. In the crystal structure, each of the two Li^I^ ions has a distorted tetra­hedral geometry, coordinated by two carboxyl­ate O atoms, one carboxyl O atom and one ethanol O atom. The carboxyl­ate group bridges the Li^I^ ions, forming a one-dimensional polymeric chain along [100]. The crystal structure is further stabilized by O—H⋯O and C—H⋯N hydrogen bonding, and π–π inter­actions with centroid–centroid distances in the range 3.6534 (13)–3.8374 (13) Å.

## Related literature

For pyrazole derivatives, see: Isloor *et al.* (2009 ▶); Skoutakis *et al.* (1988 ▶); Di Marzo *et al.* (2004 ▶); Kalluraya *et al.* (2004 ▶); Hanamoto *et al.* (2008 ▶). For a similar coordination geometry, see: Fischer (2005 ▶). For bond-length data, see: Allen *et al.* (1987 ▶). For the stability of the temperature controller used in the data collection, see: Cosier & Glazer (1986 ▶).
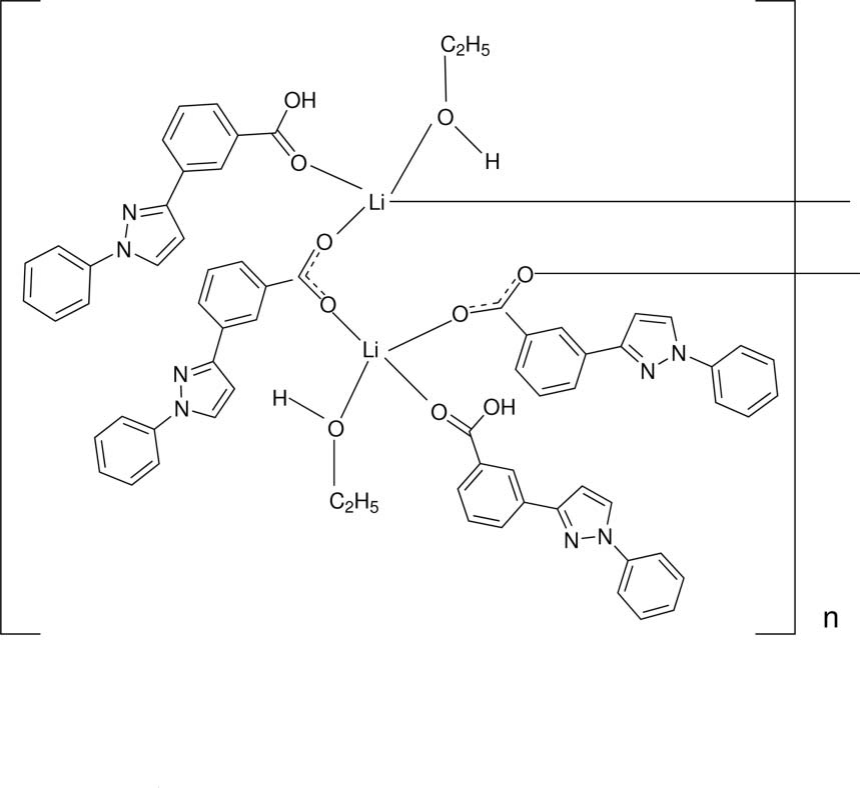


## Experimental

### 

#### Crystal data

[Li_2_(C_16_H_11_N_2_O_2_)_2_(C_16_H_12_N_2_O_2_)_2_(C_2_H_6_O)_2_]
*M* *_r_* = 1161.10
Triclinic, 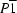

*a* = 7.9607 (1) Å
*b* = 14.9527 (2) Å
*c* = 26.5320 (4) Å
α = 103.471 (1)°
β = 90.348 (1)°
γ = 105.429 (1)°
*V* = 2952.82 (7) Å^3^
*Z* = 2
Mo *K*α radiation
μ = 0.09 mm^−1^
*T* = 100 K
0.47 × 0.18 × 0.15 mm

#### Data collection

Bruker SMART APEXII CCD area-detector diffractometer
Absorption correction: multi-scan (**SADABS**; Bruker, 2005 ▶) *T*_min_ = 0.960, *T*_max_ = 0.987
66644 measured reflections
17230 independent reflections
11897 reflections with *I* > 2σ(*I*)
*R*_int_ = 0.046

#### Refinement

*R*[*F* ^2^ > 2σ(*F* ^2^)] = 0.066
*wR*(*F* ^2^) = 0.168
*S* = 1.04
17230 reflections
811 parameters
H atoms treated by a mixture of independent and constrained refinement
Δρ_max_ = 0.41 e Å^−3^
Δρ_min_ = −0.31 e Å^−3^

### 

Data collection: *APEX2* (Bruker, 2005 ▶); cell refinement: *SAINT* (Bruker, 2005 ▶); data reduction: *SAINT*; program(s) used to solve structure: *SHELXTL* (Sheldrick, 2008 ▶); program(s) used to refine structure: *SHELXTL*; molecular graphics: *SHELXTL*; software used to prepare material for publication: *SHELXTL* and *PLATON* (Spek, 2009 ▶).

## Supplementary Material

Crystal structure: contains datablocks global, I. DOI: 10.1107/S1600536809026385/is2436sup1.cif

Structure factors: contains datablocks I. DOI: 10.1107/S1600536809026385/is2436Isup2.hkl

Additional supplementary materials:  crystallographic information; 3D view; checkCIF report

## Figures and Tables

**Table 1:** Selected bond lengths (Å)

Li1—O1*A*	1.912 (4)
Li1—O2*D*	1.931 (4)
Li1—O4	1.953 (4)
Li1—O1*C*^i^	1.954 (4)
Li2—O2*C*	1.912 (4)
Li2—O1*B*	1.931 (4)
Li2—O1*D*	1.940 (4)
Li2—O3	1.960 (4)

Symmetry code: (i) 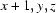.

**Table 2:** Hydrogen-bond geometry (Å, °)

*D*—H⋯*A*	*D*—H	H⋯*A*	*D*⋯*A*	*D*—H⋯*A*
O2*A*—H1*OA*⋯O2*C*^i^	0.91 (3)	1.70 (3)	2.594 (2)	167 (3)
O2*B*—H1*OB*⋯O2*D*	0.92 (3)	1.68 (3)	2.584 (2)	165 (3)
O3—H1*O*3⋯O1*C*^i^	0.85 (3)	1.92 (3)	2.761 (2)	171 (3)
O4—H1*O*4⋯O1*D*^i^	0.85 (3)	1.94 (3)	2.780 (2)	170 (2)
C7*A*—H7*AA*⋯N2*D*	0.93	2.58	3.306 (3)	136
C5*C*—H5*CA*⋯N2*B*^ii^	0.93	2.60	3.501 (3)	163

Symmetry codes: (i) 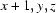; (ii) 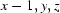.

**Fig. 1.**
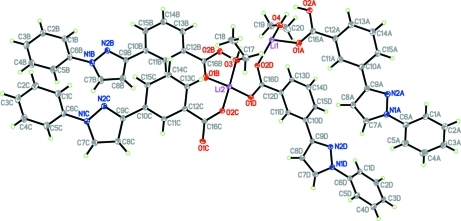
The molecular structure of (I), showing 30% probability displacement ellipsoids and the atom numbering scheme.

**Fig. 2.**
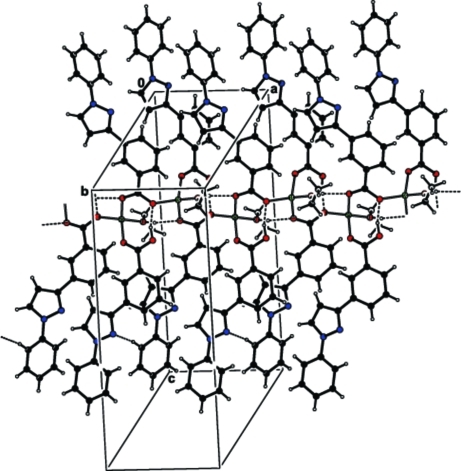
The polymeric network of (I) along the [100] direction. Dashed lines indicate the hydrogen bonding.

## supplementary crystallographic information

### Comment

Natural antibiotic compounds have become indispensable to the current health care system, assisting and complementing the natural immune system against microbial pathogens. However, because conventional antibiotics are often overused to treat microbial infections, some microorganisms have developed resistance to many of these antibiotics. Pyrazole chemistry has been a focus of medical research for more than five decades due to the versatile biological and pharmacological activities of pyrazole derivatives, including effects as antimicrobial (Isloor *et al.*, 2009), antiviral (Skoutakis *et al.*, 1988), antitumor (Di Marzo *et al.*, 2004), anti-inflammatory (Kalluraya *et al.*, 2004), antihistaminic, antifungal, anti-rheumatoid-arthritic, anticonvulsant and antipyretic (Hanamoto *et al.*, 2008) agents. We report here a lithium salt of the pyrazole derivative.

The asymmetric unit of (I) (Fig. 1), contains two Li ions, two 3-(1-phenyl-1*H*-pyrazol-3-yl)benzoate ions, two 3-(1-phenyl-1*H*-pyrazol-3-yl)benzoic acid and two ethanol molecules. The bond lengths (Allen *et al.*, 1987) and angles are normal. The torsion angles of C6A—N1A—C7A—C8A, C4A—C5A—C6A—N1A, C2A—C1A—C6A—N1A, C10A—C11A—C12A—C16A, C16A—C12A—C13A—C14A, C13A—C12A—C16A—O1A, C11A—C12A—C16A—O2A, C6B—N1B—C7B—C8B, C4B—C5B—C6B—N1B, C2B—C1B—C6B—N1B, C10B—C11B—C12B—C16B, C16B—C12B—C13B—C14B, C13B—C12B—C16B—O1B, C11B—C12B—C16B—O2B, C6C—N1C—C7C—C8C, C4C—C5C—C6C—N1C, C2C—C1C—C6C—N1C, C10C—C11C—C12C—C16C, C16C—C12C—C13C—C14C, C13C—C12C—C16C—O1C, C11C—C12C—C16C—O2C, C6D—N1D—C7D—C8D, C4D—C5D—C6D—N1D, C2D—C1D—C6D—N1D, C10D—C11D—C12D—C16D, C16D—C12D—C13D—C14D, C13D—C12D—C16D—O1D and C11D—C12D—C16D—O2D are -178.7 (2), 178.8 (2), -178.3 (2), 177.4 (2), -177.9 (2), -176.8 (2), -174.7 (2), -179.1 (2), -179.4 (2), -179.9 (2), 177.1 (2), -176.6 (2), 171.3 (2), 176.2 (2), 178.7 (2), 178.3 (2), -179.5 (2), 178.2 (2), -178.3 (2), 172.0 (2), 173.8 (2), -180.0 (2), 177.9 (2), -178.8 (2), 177.7 (2), -177.7 (2), 172.0 (2) and 174.2 (2)°, respectively. The environments of both Li ions adopt a distorted tetrahedral geometry, with each Li atom coordinated by two carboxylate oxygen atoms, one carboxyl oxygen atom and an ethanol oxygen atom (Table 1). This coordination is similar to that observed in the crystal structure of lithium hydrogen (*RS*)-phenylsuccinate (Fischer, 2005). In the crystal packing (Fig. 2), the molecules form one-dimensional polymeric chains along the [100] direction. In each chain, the ethanol hydrogen atom is hydrogen-bonded to the carboxylate oxygen atoms through O—H···O hydrogen bonding. The carboxyl hydrogen atom is also hydrogen-bonded to the carboxylate oxygen atoms *via* O—H···O hydrogen bonding. The crystal structure is further stabilized by C—H···N hydrogen-bonding (Table 2) and π–π interactions. The π–π interaction between the five-membered rings, N1A—N2A/C9A—C7A and N1B—N2B/C9B—C7B, has centroid-to-centroid distance of 3.6534 (13) Å. The π–π interactions between the six-membered rings, C1A—C6A/C10A—C15A and C1B—C6B/C10B—C15B, have centroid-to-centroid distances of between 3.7099 (14) and 3.7589 (14) Å. The π–π interaction between the five-membered and six-membered rings, N1C—N2C/C9C—C7C and C10B—C15B, has centroid-to-centroid distance of 3.8374 (13) Å.

### Experimental

The title compound is obtained by adding 3-bromo-1-phenyl pyrazole (500 mg, 2.2 mmol) into a stirred solution of benzene boronic acid (369 mg, 2.2 mmol) in toluene (10 ml) and water (10 ml). Sodium carbonate (370 mg, 4.4 mmol), and tetrakis(triphenylphosphine)palladium(0) (258 mg, 0.2 mmol) were then added. The reaction mixture was heated to 100°C for 12 hr when TLC showed completion of reaction. Reaction mixture was diluted with water, acidified to pH 3, and extracted with ethyl acetate. The combined organic layer was dried over sodium sulfate, concentrated and the residue purified by column chromatography using 5% methanol in chloroform to get pure product as colorless crystals, Yield: 510 mg (84%), Melting point: 435–438 K. The lithium is an impurity with silica gel. The NMR data is consistent with the structure of the title compound (I).

### Refinement

H atoms were positioned geometrically (C—H = 0.93–0.97 Å) and refined using a riding model with *U*_iso_(H) = 1.2*U*_eq_(C) and 1.5*U*_eq_(methyl C). A rotating–group model was used for the methyl groups. The oxygen H atoms were located from the difference Fourier map [O–H = 0.85 (3)–0.91 (3) Å] and allowed to refine freely.

### Crystal data

**Table:**

[Li_2_(C_16_H_11_N_2_O_2_)_2_(C_16_H_12_N_2_O_2_)_2_(C_2_H_6_O)_2_]	*Z* = 2
*M**_r_* = 1161.10	*F*(000) = 1216
Triclinic, *P*1	*D*_x_ = 1.306 Mg m^−^^3^
Hall symbol: -P 1	Mo *K*α radiation, λ = 0.71073 Å
*a* = 7.9607 (1) Å	Cell parameters from 9941 reflections
*b* = 14.9527 (2) Å	θ = 2.4–30.1°
*c* = 26.5320 (4) Å	µ = 0.09 mm^−^^1^
α = 103.471 (1)°	*T* = 100 K
β = 90.348 (1)°	Block, colorless
γ = 105.429 (1)°	0.47 × 0.18 × 0.15 mm
*V* = 2952.82 (7) Å^3^	

### Data collection

**Table:**

Bruker SMART APEXII CCD area-detector diffractometer	17230 independent reflections
Radiation source: fine-focus sealed tube	11897 reflections with *I* > 2σ(*I*)
graphite	*R*_int_ = 0.046
φ and ω scans	θ_max_ = 30.2°, θ_min_ = 0.8°
Absorption correction: multi-scan (*SADABS*; Bruker, 2005)	*h* = −11→11
*T*_min_ = 0.960, *T*_max_ = 0.987	*k* = −20→21
66644 measured reflections	*l* = −35→37

### Refinement

**Table:**

Refinement on *F*^2^	Primary atom site location: structure-invariant direct methods
Least-squares matrix: full	Secondary atom site location: difference Fourier map
*R*[*F*^2^ > 2σ(*F*^2^)] = 0.066	Hydrogen site location: inferred from neighbouring sites
*wR*(*F*^2^) = 0.168	H atoms treated by a mixture of independent and constrained refinement
*S* = 1.04	*w* = 1/[σ^2^(*F*_o_^2^) + (0.0531*P*)^2^ + 3.024*P*] where *P* = (*F*_o_^2^ + 2*F*_c_^2^)/3
17230 reflections	(Δ/σ)_max_ < 0.001
811 parameters	Δρ_max_ = 0.41 e Å^−^^3^
0 restraints	Δρ_min_ = −0.31 e Å^−^^3^

### Special details

**Table:**

Experimental. The crystal was placed in the cold stream of an Oxford Cryosystems Cobra open-flow nitrogen cryostat (Cosier & Glazer, 1986) operating at 100.0 (1) K.
Geometry. All s.u.'s (except the s.u. in the dihedral angle between two l.s. planes) are estimated using the full covariance matrix. The cell s.u.'s are taken into account individually in the estimation of s.u.'s in distances, angles and torsion angles; correlations between s.u.'s in cell parameters are only used when they are defined by crystal symmetry. An approximate (isotropic) treatment of cell s.u.'s is used for estimating s.u.'s involving l.s. planes.
Refinement. Refinement of F^2^ against ALL reflections. The weighted R-factor wR and goodness of fit S are based on F^2^, conventional R-factors R are based on F, with F set to zero for negative F^2^. The threshold expression of F^2^ > σ(F^2^) is used only for calculating R-factors(gt) etc. and is not relevant to the choice of reflections for refinement. R-factors based on F^2^ are statistically about twice as large as those based on F, and R- factors based on ALL data will be even larger.

### Fractional atomic coordinates and isotropic or equivalent isotropic displacement parameters (Å^2^)

**Table:**

	*x*	*y*	*z*	*U*_iso_*/*U*_eq_	
Li1	1.4484 (4)	0.4845 (2)	0.21870 (13)	0.0220 (7)	
Li2	0.9517 (4)	0.5029 (3)	0.27808 (13)	0.0224 (7)	
O1A	1.4132 (2)	0.35616 (11)	0.17852 (6)	0.0327 (4)	
O2A	1.6215 (2)	0.30183 (12)	0.20755 (6)	0.0293 (3)	
N1A	1.0252 (2)	0.06256 (12)	−0.06878 (7)	0.0243 (4)	
N2A	1.1522 (2)	0.05002 (12)	−0.03866 (6)	0.0230 (4)	
C1A	1.0440 (3)	−0.07475 (16)	−0.13585 (8)	0.0289 (5)	
H1AA	1.1124	−0.0906	−0.1126	0.035*	
C2A	1.0046 (3)	−0.13031 (18)	−0.18655 (9)	0.0347 (5)	
H2AA	1.0475	−0.1833	−0.1972	0.042*	
C3A	0.9028 (3)	−0.10783 (19)	−0.22109 (9)	0.0399 (6)	
H3AA	0.8780	−0.1449	−0.2551	0.048*	
C4A	0.8374 (4)	−0.0297 (2)	−0.20491 (10)	0.0426 (6)	
H4AA	0.7666	−0.0151	−0.2280	0.051*	
C5A	0.8762 (3)	0.02741 (18)	−0.15462 (9)	0.0359 (5)	
H5AA	0.8329	0.0803	−0.1441	0.043*	
C6A	0.9805 (3)	0.00437 (15)	−0.12024 (8)	0.0258 (4)	
C7A	0.9607 (3)	0.13500 (15)	−0.04342 (9)	0.0309 (5)	
H7AA	0.8740	0.1563	−0.0565	0.037*	
C8A	1.0462 (3)	0.17068 (15)	0.00468 (9)	0.0298 (5)	
H8AA	1.0301	0.2204	0.0309	0.036*	
C9A	1.1647 (3)	0.11558 (14)	0.00604 (8)	0.0226 (4)	
C10A	1.2922 (3)	0.12322 (15)	0.04811 (8)	0.0235 (4)	
C11A	1.3292 (3)	0.20125 (14)	0.09116 (8)	0.0225 (4)	
H11A	1.2711	0.2483	0.0937	0.027*	
C12A	1.4534 (3)	0.20890 (15)	0.13040 (8)	0.0240 (4)	
C13A	1.5398 (3)	0.13848 (17)	0.12752 (9)	0.0303 (5)	
H13A	1.6220	0.1438	0.1539	0.036*	
C14A	1.5021 (3)	0.06018 (17)	0.08493 (9)	0.0331 (5)	
H14A	1.5593	0.0128	0.0827	0.040*	
C15A	1.3794 (3)	0.05260 (16)	0.04567 (8)	0.0283 (5)	
H15A	1.3547	−0.0001	0.0173	0.034*	
C16A	1.4931 (3)	0.29600 (15)	0.17435 (8)	0.0236 (4)	
O1B	1.0385 (2)	0.63497 (11)	0.31587 (6)	0.0289 (3)	
O2B	1.3177 (2)	0.67241 (11)	0.29641 (6)	0.0277 (3)	
N1B	0.9371 (2)	0.93770 (12)	0.56028 (7)	0.0229 (4)	
N2B	1.0854 (2)	0.93899 (12)	0.53531 (6)	0.0216 (3)	
C1B	1.1010 (3)	1.06570 (16)	0.63134 (8)	0.0267 (4)	
H1BA	1.2014	1.0670	0.6135	0.032*	
C2B	1.1088 (3)	1.12830 (17)	0.67979 (8)	0.0308 (5)	
H2BA	1.2148	1.1709	0.6945	0.037*	
C3B	0.9589 (3)	1.12698 (18)	0.70596 (9)	0.0362 (6)	
H3BA	0.9640	1.1687	0.7383	0.043*	
C4B	0.8006 (3)	1.06335 (18)	0.68384 (9)	0.0361 (5)	
H4BA	0.6997	1.0633	0.7013	0.043*	
C5B	0.7918 (3)	0.99997 (16)	0.63590 (9)	0.0311 (5)	
H5BA	0.6858	0.9570	0.6214	0.037*	
C6B	0.9428 (3)	1.00136 (15)	0.60980 (8)	0.0243 (4)	
C7B	0.7971 (3)	0.87027 (16)	0.53268 (9)	0.0339 (5)	
H7BA	0.6828	0.8567	0.5424	0.041*	
C8B	0.8557 (3)	0.82614 (16)	0.48798 (9)	0.0317 (5)	
H8BA	0.7903	0.7769	0.4612	0.038*	
C9B	1.0358 (3)	0.87105 (14)	0.49110 (8)	0.0207 (4)	
C10B	1.1659 (3)	0.84961 (14)	0.45499 (8)	0.0227 (4)	
C11B	1.1187 (3)	0.78132 (14)	0.40765 (7)	0.0210 (4)	
H11B	1.0014	0.7500	0.3980	0.025*	
C12B	1.2456 (3)	0.75986 (14)	0.37487 (8)	0.0234 (4)	
C13B	1.4212 (3)	0.80507 (17)	0.38890 (9)	0.0318 (5)	
H13B	1.5058	0.7904	0.3670	0.038*	
C14B	1.4695 (3)	0.87257 (18)	0.43609 (9)	0.0374 (6)	
H14B	1.5869	0.9033	0.4458	0.045*	
C15B	1.3434 (3)	0.89403 (16)	0.46862 (9)	0.0322 (5)	
H15B	1.3773	0.9389	0.5002	0.039*	
C16B	1.1898 (3)	0.68338 (14)	0.32605 (7)	0.0215 (4)	
O1C	0.45630 (18)	0.48572 (11)	0.29247 (5)	0.0240 (3)	
O2C	0.70998 (17)	0.44937 (10)	0.28620 (5)	0.0206 (3)	
N1C	0.3491 (2)	0.71485 (12)	0.55515 (6)	0.0203 (3)	
N2C	0.4858 (2)	0.68099 (12)	0.53726 (6)	0.0206 (3)	
C1C	0.4597 (3)	0.75143 (15)	0.64512 (8)	0.0239 (4)	
H1CA	0.5194	0.7050	0.6362	0.029*	
C2C	0.4694 (3)	0.80360 (16)	0.69617 (8)	0.0290 (5)	
H2CA	0.5379	0.7929	0.7215	0.035*	
C3C	0.3778 (3)	0.87166 (16)	0.70978 (8)	0.0321 (5)	
H3CA	0.3828	0.9054	0.7442	0.039*	
C4C	0.2791 (3)	0.88917 (17)	0.67205 (9)	0.0337 (5)	
H4CA	0.2180	0.9349	0.6812	0.040*	
C5C	0.2709 (3)	0.83868 (16)	0.62051 (8)	0.0282 (5)	
H5CA	0.2063	0.8514	0.5950	0.034*	
C6C	0.3598 (3)	0.76925 (14)	0.60752 (7)	0.0214 (4)	
C7C	0.2168 (3)	0.69150 (15)	0.51775 (8)	0.0236 (4)	
H7CA	0.1113	0.7072	0.5214	0.028*	
C8C	0.2681 (3)	0.64053 (15)	0.47366 (8)	0.0229 (4)	
H8CA	0.2055	0.6145	0.4415	0.027*	
C9C	0.4369 (3)	0.63602 (14)	0.48750 (7)	0.0192 (4)	
C10C	0.5541 (2)	0.58987 (14)	0.45517 (7)	0.0185 (4)	
C11C	0.5233 (3)	0.56048 (14)	0.40109 (7)	0.0187 (4)	
H11C	0.4279	0.5709	0.3853	0.022*	
C12C	0.6337 (2)	0.51578 (13)	0.37057 (7)	0.0178 (4)	
C13C	0.7780 (3)	0.50106 (14)	0.39425 (7)	0.0197 (4)	
H13C	0.8530	0.4719	0.3742	0.024*	
C14C	0.8087 (3)	0.53019 (14)	0.44790 (8)	0.0217 (4)	
H14C	0.9046	0.5202	0.4637	0.026*	
C15C	0.6984 (3)	0.57396 (14)	0.47825 (7)	0.0206 (4)	
H15C	0.7203	0.5929	0.5142	0.025*	
C16C	0.5968 (2)	0.48180 (14)	0.31252 (7)	0.0184 (4)	
O1D	0.96510 (18)	0.49718 (11)	0.20445 (5)	0.0230 (3)	
O2D	1.25582 (18)	0.53777 (10)	0.21192 (5)	0.0205 (3)	
N1D	0.6220 (2)	0.27979 (12)	−0.05822 (6)	0.0206 (3)	
N2D	0.7938 (2)	0.31606 (12)	−0.04095 (6)	0.0200 (3)	
C1D	0.6886 (3)	0.25619 (15)	−0.14861 (8)	0.0242 (4)	
H1DA	0.7943	0.3030	−0.1389	0.029*	
C2D	0.6411 (3)	0.20990 (16)	−0.20067 (8)	0.0276 (5)	
H2DA	0.7164	0.2257	−0.2259	0.033*	
C3D	0.4833 (3)	0.14069 (16)	−0.21533 (8)	0.0292 (5)	
H3DA	0.4521	0.1107	−0.2502	0.035*	
C4D	0.3723 (3)	0.11638 (16)	−0.17781 (9)	0.0309 (5)	
H4DA	0.2660	0.0700	−0.1876	0.037*	
C5D	0.4182 (3)	0.16084 (15)	−0.12542 (8)	0.0267 (4)	
H5DA	0.3446	0.1436	−0.1002	0.032*	
C6D	0.5760 (3)	0.23139 (14)	−0.11147 (7)	0.0206 (4)	
C7D	0.5147 (3)	0.29660 (16)	−0.01962 (8)	0.0246 (4)	
H7DA	0.3932	0.2783	−0.0227	0.030*	
C8D	0.6191 (3)	0.34543 (15)	0.02464 (8)	0.0245 (4)	
H8DA	0.5838	0.3673	0.0575	0.029*	
C9D	0.7913 (3)	0.35560 (14)	0.00984 (7)	0.0194 (4)	
C10D	0.9570 (3)	0.40278 (13)	0.04207 (7)	0.0189 (4)	
C11D	0.9577 (3)	0.42900 (14)	0.09619 (7)	0.0195 (4)	
H11D	0.8527	0.4164	0.1119	0.023*	
C12D	1.1138 (3)	0.47380 (13)	0.12698 (7)	0.0180 (4)	
C13D	1.2705 (3)	0.49167 (14)	0.10350 (7)	0.0196 (4)	
H13D	1.3750	0.5209	0.1238	0.024*	
C14D	1.2716 (3)	0.46595 (14)	0.04972 (8)	0.0221 (4)	
H14D	1.3769	0.4779	0.0341	0.027*	
C15D	1.1159 (3)	0.42249 (14)	0.01917 (7)	0.0204 (4)	
H15D	1.1175	0.4064	−0.0168	0.025*	
C16D	1.1096 (3)	0.50458 (14)	0.18509 (7)	0.0189 (4)	
O3	1.1049 (2)	0.44372 (11)	0.30900 (6)	0.0254 (3)	
C17	1.0641 (3)	0.34913 (16)	0.31647 (10)	0.0338 (5)	
H17A	1.1206	0.3108	0.2912	0.041*	
H17B	0.9389	0.3204	0.3106	0.041*	
C18	1.1228 (3)	0.34828 (19)	0.37057 (11)	0.0384 (6)	
H18A	1.0993	0.2834	0.3735	0.058*	
H18B	1.0606	0.3821	0.3955	0.058*	
H18C	1.2460	0.3788	0.3770	0.058*	
O4	1.6609 (2)	0.54954 (11)	0.19115 (6)	0.0267 (3)	
C19	1.7212 (3)	0.64612 (18)	0.18609 (11)	0.0407 (6)	
H19A	1.8151	0.6818	0.2125	0.049*	
H19B	1.6266	0.6761	0.1918	0.049*	
C20	1.7857 (4)	0.6501 (2)	0.13303 (13)	0.0532 (8)	
H20A	1.8269	0.7156	0.1311	0.080*	
H20B	1.6919	0.6168	0.1069	0.080*	
H20C	1.8795	0.6205	0.1273	0.080*	
H1OA	1.642 (4)	0.357 (2)	0.2328 (11)	0.044 (8)*	
H1OB	1.276 (4)	0.623 (2)	0.2673 (12)	0.052 (9)*	
H1O3	1.215 (4)	0.460 (2)	0.3076 (11)	0.043 (8)*	
H1O4	1.750 (4)	0.5287 (19)	0.1914 (10)	0.036 (7)*	

### Atomic displacement parameters (Å^2^)

**Table:**

	*U*^11^	*U*^22^	*U*^33^	*U*^12^	*U*^13^	*U*^23^
Li1	0.0187 (17)	0.0279 (17)	0.0179 (15)	0.0079 (14)	−0.0006 (13)	0.0011 (13)
Li2	0.0185 (17)	0.0281 (17)	0.0166 (15)	0.0050 (14)	−0.0007 (13)	−0.0006 (13)
O1A	0.0382 (9)	0.0259 (8)	0.0301 (8)	0.0127 (7)	−0.0098 (7)	−0.0050 (6)
O2A	0.0300 (8)	0.0321 (8)	0.0224 (7)	0.0123 (7)	−0.0044 (6)	−0.0044 (6)
N1A	0.0245 (9)	0.0213 (8)	0.0246 (9)	0.0058 (7)	−0.0021 (7)	0.0017 (7)
N2A	0.0220 (9)	0.0242 (8)	0.0211 (8)	0.0056 (7)	−0.0005 (7)	0.0030 (7)
C1A	0.0267 (11)	0.0301 (11)	0.0251 (10)	0.0061 (9)	−0.0023 (9)	−0.0007 (8)
C2A	0.0316 (13)	0.0357 (12)	0.0303 (12)	0.0098 (10)	−0.0009 (10)	−0.0051 (10)
C3A	0.0395 (14)	0.0464 (15)	0.0256 (11)	0.0085 (12)	−0.0054 (10)	−0.0034 (10)
C4A	0.0431 (15)	0.0504 (16)	0.0320 (13)	0.0112 (12)	−0.0103 (11)	0.0076 (11)
C5A	0.0381 (14)	0.0358 (12)	0.0332 (12)	0.0129 (11)	−0.0050 (10)	0.0045 (10)
C6A	0.0246 (11)	0.0262 (10)	0.0229 (10)	0.0038 (8)	−0.0013 (8)	0.0026 (8)
C7A	0.0307 (12)	0.0232 (10)	0.0357 (12)	0.0102 (9)	−0.0069 (9)	−0.0018 (9)
C8A	0.0321 (12)	0.0216 (10)	0.0326 (11)	0.0101 (9)	−0.0036 (9)	−0.0022 (8)
C9A	0.0234 (10)	0.0187 (9)	0.0233 (10)	0.0035 (8)	0.0016 (8)	0.0030 (7)
C10A	0.0225 (10)	0.0231 (9)	0.0213 (9)	0.0037 (8)	0.0021 (8)	0.0016 (8)
C11A	0.0230 (10)	0.0204 (9)	0.0228 (10)	0.0053 (8)	0.0028 (8)	0.0035 (7)
C12A	0.0261 (11)	0.0242 (10)	0.0195 (9)	0.0066 (8)	0.0034 (8)	0.0012 (8)
C13A	0.0321 (12)	0.0346 (12)	0.0241 (10)	0.0140 (10)	−0.0011 (9)	0.0014 (9)
C14A	0.0380 (13)	0.0318 (11)	0.0304 (11)	0.0181 (10)	−0.0006 (10)	−0.0002 (9)
C15A	0.0320 (12)	0.0280 (11)	0.0221 (10)	0.0104 (9)	0.0017 (9)	−0.0019 (8)
C16A	0.0233 (10)	0.0242 (10)	0.0196 (9)	0.0041 (8)	0.0014 (8)	0.0010 (8)
O1B	0.0251 (8)	0.0263 (7)	0.0276 (8)	0.0035 (6)	0.0030 (6)	−0.0043 (6)
O2B	0.0231 (8)	0.0327 (8)	0.0204 (7)	0.0054 (6)	0.0029 (6)	−0.0048 (6)
N1B	0.0251 (9)	0.0195 (8)	0.0227 (8)	0.0053 (7)	0.0074 (7)	0.0029 (6)
N2B	0.0222 (9)	0.0241 (8)	0.0192 (8)	0.0095 (7)	0.0041 (6)	0.0032 (6)
C1B	0.0275 (11)	0.0304 (11)	0.0211 (10)	0.0098 (9)	0.0046 (8)	0.0016 (8)
C2B	0.0313 (12)	0.0313 (11)	0.0255 (11)	0.0081 (9)	0.0004 (9)	−0.0009 (9)
C3B	0.0437 (15)	0.0368 (13)	0.0235 (11)	0.0119 (11)	0.0053 (10)	−0.0024 (9)
C4B	0.0372 (14)	0.0394 (13)	0.0286 (12)	0.0092 (11)	0.0128 (10)	0.0034 (10)
C5B	0.0303 (12)	0.0289 (11)	0.0296 (11)	0.0041 (9)	0.0070 (9)	0.0031 (9)
C6B	0.0311 (11)	0.0231 (10)	0.0194 (9)	0.0102 (8)	0.0064 (8)	0.0034 (8)
C7B	0.0254 (12)	0.0266 (11)	0.0382 (13)	−0.0021 (9)	0.0107 (10)	−0.0038 (9)
C8B	0.0275 (12)	0.0229 (10)	0.0341 (12)	−0.0007 (9)	0.0057 (9)	−0.0047 (9)
C9B	0.0226 (10)	0.0184 (9)	0.0210 (9)	0.0071 (8)	0.0019 (8)	0.0030 (7)
C10B	0.0243 (10)	0.0228 (9)	0.0194 (9)	0.0069 (8)	0.0019 (8)	0.0014 (7)
C11B	0.0214 (10)	0.0199 (9)	0.0197 (9)	0.0047 (7)	−0.0008 (7)	0.0024 (7)
C12B	0.0239 (10)	0.0223 (9)	0.0207 (9)	0.0061 (8)	0.0001 (8)	−0.0008 (7)
C13B	0.0254 (11)	0.0345 (12)	0.0290 (11)	0.0076 (9)	0.0045 (9)	−0.0039 (9)
C14B	0.0215 (11)	0.0407 (13)	0.0360 (13)	0.0020 (10)	−0.0008 (9)	−0.0102 (10)
C15B	0.0282 (12)	0.0311 (11)	0.0266 (11)	0.0041 (9)	−0.0008 (9)	−0.0093 (9)
C16B	0.0241 (10)	0.0214 (9)	0.0185 (9)	0.0084 (8)	0.0001 (8)	0.0014 (7)
O1C	0.0165 (7)	0.0393 (8)	0.0175 (7)	0.0114 (6)	0.0007 (5)	0.0052 (6)
O2C	0.0157 (7)	0.0264 (7)	0.0176 (6)	0.0064 (6)	0.0009 (5)	0.0007 (5)
N1C	0.0202 (8)	0.0228 (8)	0.0174 (8)	0.0067 (7)	0.0020 (6)	0.0031 (6)
N2C	0.0193 (8)	0.0210 (8)	0.0199 (8)	0.0051 (6)	0.0015 (6)	0.0024 (6)
C1C	0.0256 (11)	0.0228 (9)	0.0217 (10)	0.0061 (8)	0.0021 (8)	0.0027 (8)
C2C	0.0322 (12)	0.0306 (11)	0.0204 (10)	0.0044 (9)	−0.0014 (9)	0.0039 (8)
C3C	0.0423 (14)	0.0295 (11)	0.0196 (10)	0.0091 (10)	0.0031 (9)	−0.0029 (8)
C4C	0.0437 (14)	0.0286 (11)	0.0289 (11)	0.0163 (10)	0.0039 (10)	−0.0002 (9)
C5C	0.0353 (12)	0.0278 (11)	0.0228 (10)	0.0126 (9)	0.0016 (9)	0.0038 (8)
C6C	0.0227 (10)	0.0210 (9)	0.0169 (9)	0.0032 (8)	0.0029 (7)	0.0011 (7)
C7C	0.0232 (10)	0.0280 (10)	0.0217 (9)	0.0104 (8)	0.0012 (8)	0.0063 (8)
C8C	0.0230 (10)	0.0276 (10)	0.0183 (9)	0.0090 (8)	0.0004 (8)	0.0037 (8)
C9C	0.0204 (10)	0.0195 (9)	0.0167 (8)	0.0039 (7)	0.0012 (7)	0.0040 (7)
C10C	0.0163 (9)	0.0211 (9)	0.0168 (8)	0.0031 (7)	0.0013 (7)	0.0045 (7)
C11C	0.0159 (9)	0.0220 (9)	0.0176 (9)	0.0047 (7)	−0.0006 (7)	0.0042 (7)
C12C	0.0167 (9)	0.0194 (8)	0.0158 (8)	0.0034 (7)	−0.0003 (7)	0.0036 (7)
C13C	0.0175 (9)	0.0227 (9)	0.0183 (9)	0.0055 (7)	0.0017 (7)	0.0039 (7)
C14C	0.0181 (10)	0.0259 (10)	0.0217 (9)	0.0061 (8)	−0.0029 (7)	0.0068 (8)
C15C	0.0199 (10)	0.0239 (9)	0.0159 (8)	0.0033 (8)	−0.0012 (7)	0.0041 (7)
C16C	0.0152 (9)	0.0217 (9)	0.0173 (9)	0.0037 (7)	0.0009 (7)	0.0045 (7)
O1D	0.0169 (7)	0.0365 (8)	0.0163 (6)	0.0113 (6)	0.0010 (5)	0.0033 (6)
O2D	0.0168 (7)	0.0282 (7)	0.0159 (6)	0.0095 (6)	−0.0008 (5)	0.0003 (5)
N1D	0.0192 (8)	0.0234 (8)	0.0171 (8)	0.0048 (7)	−0.0004 (6)	0.0021 (6)
N2D	0.0189 (8)	0.0212 (8)	0.0185 (8)	0.0065 (6)	−0.0015 (6)	0.0014 (6)
C1D	0.0240 (10)	0.0238 (10)	0.0221 (10)	0.0058 (8)	0.0006 (8)	0.0008 (8)
C2D	0.0335 (12)	0.0306 (11)	0.0188 (9)	0.0116 (9)	0.0049 (8)	0.0026 (8)
C3D	0.0334 (12)	0.0318 (11)	0.0185 (9)	0.0116 (9)	−0.0038 (8)	−0.0044 (8)
C4D	0.0288 (12)	0.0283 (11)	0.0270 (11)	0.0015 (9)	−0.0039 (9)	−0.0023 (9)
C5D	0.0267 (11)	0.0262 (10)	0.0232 (10)	0.0033 (9)	0.0003 (8)	0.0029 (8)
C6D	0.0229 (10)	0.0220 (9)	0.0164 (9)	0.0076 (8)	−0.0018 (7)	0.0019 (7)
C7D	0.0191 (10)	0.0329 (11)	0.0202 (9)	0.0063 (8)	0.0022 (8)	0.0041 (8)
C8D	0.0228 (10)	0.0299 (10)	0.0189 (9)	0.0067 (8)	0.0019 (8)	0.0032 (8)
C9D	0.0225 (10)	0.0202 (9)	0.0158 (8)	0.0076 (7)	0.0003 (7)	0.0028 (7)
C10D	0.0205 (10)	0.0196 (9)	0.0160 (8)	0.0074 (7)	−0.0009 (7)	0.0012 (7)
C11D	0.0181 (9)	0.0227 (9)	0.0176 (9)	0.0071 (7)	0.0010 (7)	0.0030 (7)
C12D	0.0193 (9)	0.0196 (9)	0.0155 (8)	0.0081 (7)	−0.0004 (7)	0.0020 (7)
C13D	0.0174 (9)	0.0227 (9)	0.0178 (9)	0.0062 (7)	0.0007 (7)	0.0023 (7)
C14D	0.0194 (10)	0.0263 (10)	0.0206 (9)	0.0075 (8)	0.0052 (7)	0.0043 (8)
C15D	0.0233 (10)	0.0235 (9)	0.0151 (8)	0.0092 (8)	0.0026 (7)	0.0026 (7)
C16D	0.0189 (9)	0.0223 (9)	0.0165 (8)	0.0090 (7)	0.0007 (7)	0.0028 (7)
O3	0.0161 (7)	0.0327 (8)	0.0292 (8)	0.0069 (6)	0.0020 (6)	0.0104 (6)
C17	0.0301 (12)	0.0253 (11)	0.0423 (13)	0.0059 (9)	−0.0002 (10)	0.0032 (10)
C18	0.0286 (12)	0.0413 (14)	0.0541 (16)	0.0120 (10)	0.0023 (11)	0.0261 (12)
O4	0.0189 (8)	0.0343 (8)	0.0309 (8)	0.0116 (6)	0.0037 (6)	0.0104 (6)
C19	0.0317 (13)	0.0292 (12)	0.0580 (17)	0.0092 (10)	0.0030 (12)	0.0033 (11)
C20	0.0403 (16)	0.0542 (17)	0.085 (2)	0.0220 (14)	0.0269 (15)	0.0441 (17)

### Geometric parameters (Å, °)

**Table:**

Li1—O1A	1.912 (4)	N1C—N2C	1.361 (2)
Li1—O2D	1.931 (4)	N1C—C6C	1.427 (2)
Li1—O4	1.953 (4)	N2C—C9C	1.337 (2)
Li1—O1C^i^	1.954 (4)	C1C—C2C	1.387 (3)
Li2—O2C	1.912 (4)	C1C—C6C	1.388 (3)
Li2—O1B	1.931 (4)	C1C—H1CA	0.9300
Li2—O1D	1.940 (4)	C2C—C3C	1.387 (3)
Li2—O3	1.960 (4)	C2C—H2CA	0.9300
O1A—C16A	1.218 (3)	C3C—C4C	1.382 (3)
O2A—C16A	1.317 (3)	C3C—H3CA	0.9300
O2A—H1OA	0.91 (3)	C4C—C5C	1.391 (3)
N1A—C7A	1.360 (3)	C4C—H4CA	0.9300
N1A—N2A	1.363 (2)	C5C—C6C	1.387 (3)
N1A—C6A	1.423 (3)	C5C—H5CA	0.9300
N2A—C9A	1.335 (2)	C7C—C8C	1.368 (3)
C1A—C6A	1.385 (3)	C7C—H7CA	0.9300
C1A—C2A	1.390 (3)	C8C—C9C	1.414 (3)
C1A—H1AA	0.9300	C8C—H8CA	0.9300
C2A—C3A	1.375 (4)	C9C—C10C	1.469 (3)
C2A—H2AA	0.9300	C10C—C15C	1.398 (3)
C3A—C4A	1.383 (4)	C10C—C11C	1.399 (3)
C3A—H3AA	0.9300	C11C—C12C	1.394 (3)
C4A—C5A	1.389 (3)	C11C—H11C	0.9300
C4A—H4AA	0.9300	C12C—C13C	1.398 (3)
C5A—C6A	1.389 (3)	C12C—C16C	1.506 (3)
C5A—H5AA	0.9300	C13C—C14C	1.388 (3)
C7A—C8A	1.362 (3)	C13C—H13C	0.9300
C7A—H7AA	0.9300	C14C—C15C	1.385 (3)
C8A—C9A	1.413 (3)	C14C—H14C	0.9300
C8A—H8AA	0.9300	C15C—H15C	0.9300
C9A—C10A	1.468 (3)	O1D—C16D	1.251 (2)
C10A—C11A	1.395 (3)	O2D—C16D	1.275 (2)
C10A—C15A	1.400 (3)	N1D—C7D	1.359 (3)
C11A—C12A	1.395 (3)	N1D—N2D	1.363 (2)
C11A—H11A	0.9300	N1D—C6D	1.425 (2)
C12A—C13A	1.391 (3)	N2D—C9D	1.342 (2)
C12A—C16A	1.492 (3)	C1D—C6D	1.384 (3)
C13A—C14A	1.389 (3)	C1D—C2D	1.392 (3)
C13A—H13A	0.9300	C1D—H1DA	0.9300
C14A—C15A	1.388 (3)	C2D—C3D	1.384 (3)
C14A—H14A	0.9300	C2D—H2DA	0.9300
C15A—H15A	0.9300	C3D—C4D	1.383 (3)
O1B—C16B	1.220 (3)	C3D—H3DA	0.9300
O2B—C16B	1.312 (2)	C4D—C5D	1.394 (3)
O2B—H1OB	0.92 (3)	C4D—H4DA	0.9300
N1B—N2B	1.356 (2)	C5D—C6D	1.391 (3)
N1B—C7B	1.357 (3)	C5D—H5DA	0.9300
N1B—C6B	1.424 (2)	C7D—C8D	1.367 (3)
N2B—C9B	1.338 (2)	C7D—H7DA	0.9300
C1B—C6B	1.388 (3)	C8D—C9D	1.408 (3)
C1B—C2B	1.393 (3)	C8D—H8DA	0.9300
C1B—H1BA	0.9300	C9D—C10D	1.475 (3)
C2B—C3B	1.382 (3)	C10D—C15D	1.397 (3)
C2B—H2BA	0.9300	C10D—C11D	1.397 (3)
C3B—C4B	1.390 (4)	C11D—C12D	1.397 (3)
C3B—H3BA	0.9300	C11D—H11D	0.9300
C4B—C5B	1.387 (3)	C12D—C13D	1.388 (3)
C4B—H4BA	0.9300	C12D—C16D	1.506 (3)
C5B—C6B	1.389 (3)	C13D—C14D	1.389 (3)
C5B—H5BA	0.9300	C13D—H13D	0.9300
C7B—C8B	1.366 (3)	C14D—C15D	1.389 (3)
C7B—H7BA	0.9300	C14D—H14D	0.9300
C8B—C9B	1.405 (3)	C15D—H15D	0.9300
C8B—H8BA	0.9300	O3—C17	1.426 (3)
C9B—C10B	1.463 (3)	O3—H1O3	0.85 (3)
C10B—C15B	1.396 (3)	C17—C18	1.511 (3)
C10B—C11B	1.398 (3)	C17—H17A	0.9700
C11B—C12B	1.390 (3)	C17—H17B	0.9700
C11B—H11B	0.9300	C18—H18A	0.9600
C12B—C13B	1.387 (3)	C18—H18B	0.9600
C12B—C16B	1.488 (3)	C18—H18C	0.9600
C13B—C14B	1.390 (3)	O4—C19	1.434 (3)
C13B—H13B	0.9300	O4—H1O4	0.85 (3)
C14B—C15B	1.381 (3)	C19—C20	1.510 (4)
C14B—H14B	0.9300	C19—H19A	0.9700
C15B—H15B	0.9300	C19—H19B	0.9700
O1C—C16C	1.257 (2)	C20—H20A	0.9600
O1C—Li1^ii^	1.954 (4)	C20—H20B	0.9600
O2C—C16C	1.272 (2)	C20—H20C	0.9600
N1C—C7C	1.359 (3)		
			
O1A—Li1—O2D	114.37 (17)	C1C—C2C—C3C	120.5 (2)
O1A—Li1—O4	100.80 (17)	C1C—C2C—H2CA	119.8
O2D—Li1—O4	113.07 (19)	C3C—C2C—H2CA	119.8
O1A—Li1—O1C^i^	109.10 (19)	C4C—C3C—C2C	119.8 (2)
O2D—Li1—O1C^i^	102.01 (16)	C4C—C3C—H3CA	120.1
O4—Li1—O1C^i^	118.00 (17)	C2C—C3C—H3CA	120.1
O2C—Li2—O1B	112.91 (18)	C3C—C4C—C5C	120.3 (2)
O2C—Li2—O1D	105.03 (16)	C3C—C4C—H4CA	119.8
O1B—Li2—O1D	108.16 (19)	C5C—C4C—H4CA	119.8
O2C—Li2—O3	112.76 (19)	C6C—C5C—C4C	119.4 (2)
O1B—Li2—O3	101.30 (16)	C6C—C5C—H5CA	120.3
O1D—Li2—O3	116.84 (18)	C4C—C5C—H5CA	120.3
C16A—O2A—H1OA	110.3 (18)	C5C—C6C—C1C	120.70 (18)
C7A—N1A—N2A	111.37 (17)	C5C—C6C—N1C	120.26 (18)
C7A—N1A—C6A	128.98 (18)	C1C—C6C—N1C	119.04 (18)
N2A—N1A—C6A	119.62 (17)	N1C—C7C—C8C	107.01 (18)
C9A—N2A—N1A	104.87 (16)	N1C—C7C—H7CA	126.5
C6A—C1A—C2A	119.4 (2)	C8C—C7C—H7CA	126.5
C6A—C1A—H1AA	120.3	C7C—C8C—C9C	105.17 (17)
C2A—C1A—H1AA	120.3	C7C—C8C—H8CA	127.4
C3A—C2A—C1A	120.7 (2)	C9C—C8C—H8CA	127.4
C3A—C2A—H2AA	119.6	N2C—C9C—C8C	111.09 (17)
C1A—C2A—H2AA	119.6	N2C—C9C—C10C	120.22 (17)
C2A—C3A—C4A	119.4 (2)	C8C—C9C—C10C	128.69 (17)
C2A—C3A—H3AA	120.3	C15C—C10C—C11C	118.81 (18)
C4A—C3A—H3AA	120.3	C15C—C10C—C9C	120.20 (17)
C3A—C4A—C5A	120.9 (2)	C11C—C10C—C9C	120.99 (17)
C3A—C4A—H4AA	119.5	C12C—C11C—C10C	120.83 (18)
C5A—C4A—H4AA	119.5	C12C—C11C—H11C	119.6
C4A—C5A—C6A	119.0 (2)	C10C—C11C—H11C	119.6
C4A—C5A—H5AA	120.5	C11C—C12C—C13C	119.60 (17)
C6A—C5A—H5AA	120.5	C11C—C12C—C16C	120.53 (17)
C1A—C6A—C5A	120.4 (2)	C13C—C12C—C16C	119.87 (17)
C1A—C6A—N1A	119.27 (19)	C14C—C13C—C12C	119.65 (18)
C5A—C6A—N1A	120.3 (2)	C14C—C13C—H13C	120.2
N1A—C7A—C8A	107.58 (19)	C12C—C13C—H13C	120.2
N1A—C7A—H7AA	126.2	C15C—C14C—C13C	120.75 (18)
C8A—C7A—H7AA	126.2	C15C—C14C—H14C	119.6
C7A—C8A—C9A	104.95 (19)	C13C—C14C—H14C	119.6
C7A—C8A—H8AA	127.5	C14C—C15C—C10C	120.37 (18)
C9A—C8A—H8AA	127.5	C14C—C15C—H15C	119.8
N2A—C9A—C8A	111.22 (18)	C10C—C15C—H15C	119.8
N2A—C9A—C10A	119.82 (18)	O1C—C16C—O2C	123.21 (17)
C8A—C9A—C10A	128.95 (18)	O1C—C16C—C12C	119.02 (17)
C11A—C10A—C15A	118.78 (19)	O2C—C16C—C12C	117.76 (16)
C11A—C10A—C9A	120.57 (19)	C7D—N1D—N2D	111.97 (15)
C15A—C10A—C9A	120.65 (18)	C7D—N1D—C6D	128.50 (17)
C10A—C11A—C12A	120.12 (19)	N2D—N1D—C6D	119.53 (16)
C10A—C11A—H11A	119.9	C9D—N2D—N1D	104.39 (16)
C12A—C11A—H11A	119.9	C6D—C1D—C2D	119.0 (2)
C13A—C12A—C11A	120.64 (19)	C6D—C1D—H1DA	120.5
C13A—C12A—C16A	121.64 (19)	C2D—C1D—H1DA	120.5
C11A—C12A—C16A	117.70 (19)	C3D—C2D—C1D	120.8 (2)
C14A—C13A—C12A	119.4 (2)	C3D—C2D—H2DA	119.6
C14A—C13A—H13A	120.3	C1D—C2D—H2DA	119.6
C12A—C13A—H13A	120.3	C4D—C3D—C2D	119.66 (19)
C15A—C14A—C13A	120.1 (2)	C4D—C3D—H3DA	120.2
C15A—C14A—H14A	120.0	C2D—C3D—H3DA	120.2
C13A—C14A—H14A	120.0	C3D—C4D—C5D	120.5 (2)
C14A—C15A—C10A	120.93 (19)	C3D—C4D—H4DA	119.7
C14A—C15A—H15A	119.5	C5D—C4D—H4DA	119.7
C10A—C15A—H15A	119.5	C6D—C5D—C4D	119.0 (2)
O1A—C16A—O2A	123.60 (18)	C6D—C5D—H5DA	120.5
O1A—C16A—C12A	122.29 (19)	C4D—C5D—H5DA	120.5
O2A—C16A—C12A	114.10 (18)	C1D—C6D—C5D	121.04 (18)
C16B—O2B—H1OB	109.8 (19)	C1D—C6D—N1D	119.29 (18)
N2B—N1B—C7B	111.66 (17)	C5D—C6D—N1D	119.67 (19)
N2B—N1B—C6B	120.00 (17)	N1D—C7D—C8D	107.07 (18)
C7B—N1B—C6B	128.33 (19)	N1D—C7D—H7DA	126.5
C9B—N2B—N1B	104.95 (16)	C8D—C7D—H7DA	126.5
C6B—C1B—C2B	119.8 (2)	C7D—C8D—C9D	105.26 (18)
C6B—C1B—H1BA	120.1	C7D—C8D—H8DA	127.4
C2B—C1B—H1BA	120.1	C9D—C8D—H8DA	127.4
C3B—C2B—C1B	119.9 (2)	N2D—C9D—C8D	111.31 (17)
C3B—C2B—H2BA	120.0	N2D—C9D—C10D	119.98 (18)
C1B—C2B—H2BA	120.0	C8D—C9D—C10D	128.71 (17)
C2B—C3B—C4B	120.0 (2)	C15D—C10D—C11D	118.67 (17)
C2B—C3B—H3BA	120.0	C15D—C10D—C9D	120.79 (17)
C4B—C3B—H3BA	120.0	C11D—C10D—C9D	120.54 (18)
C5B—C4B—C3B	120.5 (2)	C12D—C11D—C10D	120.91 (18)
C5B—C4B—H4BA	119.7	C12D—C11D—H11D	119.5
C3B—C4B—H4BA	119.7	C10D—C11D—H11D	119.5
C4B—C5B—C6B	119.3 (2)	C13D—C12D—C11D	119.53 (17)
C4B—C5B—H5BA	120.3	C13D—C12D—C16D	120.82 (17)
C6B—C5B—H5BA	120.3	C11D—C12D—C16D	119.63 (17)
C1B—C6B—C5B	120.43 (19)	C12D—C13D—C14D	120.13 (18)
C1B—C6B—N1B	119.14 (19)	C12D—C13D—H13D	119.9
C5B—C6B—N1B	120.42 (19)	C14D—C13D—H13D	119.9
N1B—C7B—C8B	107.2 (2)	C15D—C14D—C13D	120.21 (19)
N1B—C7B—H7BA	126.4	C15D—C14D—H14D	119.9
C8B—C7B—H7BA	126.4	C13D—C14D—H14D	119.9
C7B—C8B—C9B	105.23 (19)	C14D—C15D—C10D	120.55 (18)
C7B—C8B—H8BA	127.4	C14D—C15D—H15D	119.7
C9B—C8B—H8BA	127.4	C10D—C15D—H15D	119.7
N2B—C9B—C8B	111.01 (18)	O1D—C16D—O2D	123.56 (17)
N2B—C9B—C10B	119.97 (18)	O1D—C16D—C12D	119.06 (17)
C8B—C9B—C10B	128.95 (18)	O2D—C16D—C12D	117.38 (17)
C15B—C10B—C11B	118.17 (19)	C17—O3—H1O3	105 (2)
C15B—C10B—C9B	120.05 (18)	Li2—O3—H1O3	123 (2)
C11B—C10B—C9B	121.70 (19)	O3—C17—C18	111.84 (19)
C12B—C11B—C10B	120.53 (19)	O3—C17—H17A	109.2
C12B—C11B—H11B	119.7	C18—C17—H17A	109.2
C10B—C11B—H11B	119.7	O3—C17—H17B	109.2
C13B—C12B—C11B	120.52 (18)	C18—C17—H17B	109.2
C13B—C12B—C16B	120.77 (19)	H17A—C17—H17B	107.9
C11B—C12B—C16B	118.61 (18)	C17—C18—H18A	109.5
C12B—C13B—C14B	119.3 (2)	C17—C18—H18B	109.5
C12B—C13B—H13B	120.3	H18A—C18—H18B	109.5
C14B—C13B—H13B	120.3	C17—C18—H18C	109.5
C15B—C14B—C13B	120.1 (2)	H18A—C18—H18C	109.5
C15B—C14B—H14B	119.9	H18B—C18—H18C	109.5
C13B—C14B—H14B	119.9	C19—O4—H1O4	106.9 (19)
C14B—C15B—C10B	121.29 (19)	Li1—O4—H1O4	118.8 (19)
C14B—C15B—H15B	119.4	O4—C19—C20	111.6 (2)
C10B—C15B—H15B	119.4	O4—C19—H19A	109.3
O1B—C16B—O2B	124.17 (18)	C20—C19—H19A	109.3
O1B—C16B—C12B	121.93 (19)	O4—C19—H19B	109.3
O2B—C16B—C12B	113.89 (18)	C20—C19—H19B	109.3
C7C—N1C—N2C	111.96 (16)	H19A—C19—H19B	108.0
C7C—N1C—C6C	128.46 (17)	C19—C20—H20A	109.5
N2C—N1C—C6C	119.58 (16)	C19—C20—H20B	109.5
C9C—N2C—N1C	104.77 (16)	H20A—C20—H20B	109.5
C2C—C1C—C6C	119.3 (2)	C19—C20—H20C	109.5
C2C—C1C—H1CA	120.4	H20A—C20—H20C	109.5
C6C—C1C—H1CA	120.4	H20B—C20—H20C	109.5
			
O2D—Li1—O1A—C16A	−174.1 (2)	N2C—N1C—C6C—C1C	−29.7 (3)
O4—Li1—O1A—C16A	64.3 (3)	N2C—N1C—C7C—C8C	−0.1 (2)
O1C^i^—Li1—O1A—C16A	−60.6 (3)	C6C—N1C—C7C—C8C	178.71 (19)
C16C^i^—Li1—O1A—C16A	−36.5 (3)	N1C—C7C—C8C—C9C	−0.2 (2)
C7A—N1A—N2A—C9A	0.4 (2)	N1C—N2C—C9C—C8C	−0.4 (2)
C6A—N1A—N2A—C9A	178.84 (18)	N1C—N2C—C9C—C10C	179.91 (17)
C6A—C1A—C2A—C3A	−0.4 (4)	C7C—C8C—C9C—N2C	0.4 (2)
C1A—C2A—C3A—C4A	−0.8 (4)	C7C—C8C—C9C—C10C	−180.0 (2)
C2A—C3A—C4A—C5A	1.3 (4)	N2C—C9C—C10C—C15C	14.1 (3)
C3A—C4A—C5A—C6A	−0.7 (4)	C8C—C9C—C10C—C15C	−165.4 (2)
C2A—C1A—C6A—C5A	0.9 (4)	N2C—C9C—C10C—C11C	−166.31 (19)
C2A—C1A—C6A—N1A	−178.3 (2)	C8C—C9C—C10C—C11C	14.1 (3)
C4A—C5A—C6A—C1A	−0.4 (4)	C15C—C10C—C11C—C12C	0.2 (3)
C4A—C5A—C6A—N1A	178.8 (2)	C9C—C10C—C11C—C12C	−179.32 (18)
C7A—N1A—C6A—C1A	−170.6 (2)	C10C—C11C—C12C—C13C	−0.7 (3)
N2A—N1A—C6A—C1A	11.3 (3)	C10C—C11C—C12C—C16C	178.23 (18)
C7A—N1A—C6A—C5A	10.1 (4)	C11C—C12C—C13C—C14C	0.7 (3)
N2A—N1A—C6A—C5A	−168.0 (2)	C16C—C12C—C13C—C14C	−178.26 (18)
N2A—N1A—C7A—C8A	−0.4 (3)	C12C—C13C—C14C—C15C	−0.2 (3)
C6A—N1A—C7A—C8A	−178.7 (2)	C13C—C14C—C15C—C10C	−0.2 (3)
N1A—C7A—C8A—C9A	0.3 (3)	C11C—C10C—C15C—C14C	0.2 (3)
N1A—N2A—C9A—C8A	−0.2 (2)	C9C—C10C—C15C—C14C	179.79 (18)
N1A—N2A—C9A—C10A	−179.50 (18)	Li1^ii^—O1C—C16C—O2C	−21.6 (3)
C7A—C8A—C9A—N2A	0.0 (3)	Li1^ii^—O1C—C16C—C12C	159.17 (18)
C7A—C8A—C9A—C10A	179.2 (2)	Li2—O2C—C16C—O1C	121.9 (2)
N2A—C9A—C10A—C11A	168.5 (2)	Li2—O2C—C16C—C12C	−58.9 (3)
C8A—C9A—C10A—C11A	−10.6 (4)	Li2—O2C—C16C—Li1^ii^	108.4 (2)
N2A—C9A—C10A—C15A	−11.2 (3)	C11C—C12C—C16C—O1C	−6.9 (3)
C8A—C9A—C10A—C15A	169.7 (2)	C13C—C12C—C16C—O1C	172.00 (19)
C15A—C10A—C11A—C12A	1.1 (3)	C11C—C12C—C16C—O2C	173.83 (18)
C9A—C10A—C11A—C12A	−178.6 (2)	C13C—C12C—C16C—O2C	−7.3 (3)
C10A—C11A—C12A—C13A	−0.9 (3)	C11C—C12C—C16C—Li1^ii^	22.7 (4)
C10A—C11A—C12A—C16A	177.42 (19)	C13C—C12C—C16C—Li1^ii^	−158.4 (3)
C11A—C12A—C13A—C14A	0.3 (4)	O2C—Li2—O1D—C16D	−162.18 (17)
C16A—C12A—C13A—C14A	−177.9 (2)	O1B—Li2—O1D—C16D	77.0 (2)
C12A—C13A—C14A—C15A	0.0 (4)	O3—Li2—O1D—C16D	−36.4 (3)
C13A—C14A—C15A—C10A	0.2 (4)	O1A—Li1—O2D—C16D	−1.3 (3)
C11A—C10A—C15A—C14A	−0.8 (3)	O4—Li1—O2D—C16D	113.3 (2)
C9A—C10A—C15A—C14A	178.9 (2)	O1C^i^—Li1—O2D—C16D	−118.9 (2)
Li1—O1A—C16A—O2A	14.2 (4)	C16C^i^—Li1—O2D—C16D	−128.7 (2)
Li1—O1A—C16A—C12A	−165.4 (2)	C7D—N1D—N2D—C9D	0.4 (2)
C13A—C12A—C16A—O1A	−176.8 (2)	C6D—N1D—N2D—C9D	−179.65 (17)
C11A—C12A—C16A—O1A	4.9 (3)	C6D—C1D—C2D—C3D	0.5 (3)
C13A—C12A—C16A—O2A	3.6 (3)	C1D—C2D—C3D—C4D	−0.8 (3)
C11A—C12A—C16A—O2A	−174.68 (19)	C2D—C3D—C4D—C5D	−0.2 (4)
O2C—Li2—O1B—C16B	165.81 (19)	C3D—C4D—C5D—C6D	1.3 (3)
O1D—Li2—O1B—C16B	−78.4 (3)	C2D—C1D—C6D—C5D	0.6 (3)
O3—Li2—O1B—C16B	45.0 (3)	C2D—C1D—C6D—N1D	−178.75 (19)
C16D—Li2—O1B—C16B	−55.5 (2)	C4D—C5D—C6D—C1D	−1.5 (3)
C7B—N1B—N2B—C9B	0.3 (2)	C4D—C5D—C6D—N1D	177.85 (19)
C6B—N1B—N2B—C9B	179.24 (18)	C7D—N1D—C6D—C1D	151.3 (2)
C6B—C1B—C2B—C3B	−0.8 (4)	N2D—N1D—C6D—C1D	−28.6 (3)
C1B—C2B—C3B—C4B	−0.1 (4)	C7D—N1D—C6D—C5D	−28.1 (3)
C2B—C3B—C4B—C5B	0.9 (4)	N2D—N1D—C6D—C5D	152.06 (19)
C3B—C4B—C5B—C6B	−0.7 (4)	N2D—N1D—C7D—C8D	−0.1 (2)
C2B—C1B—C6B—C5B	1.0 (3)	C6D—N1D—C7D—C8D	−179.99 (19)
C2B—C1B—C6B—N1B	−179.9 (2)	N1D—C7D—C8D—C9D	−0.3 (2)
C4B—C5B—C6B—C1B	−0.2 (4)	N1D—N2D—C9D—C8D	−0.6 (2)
C4B—C5B—C6B—N1B	−179.4 (2)	N1D—N2D—C9D—C10D	180.00 (17)
N2B—N1B—C6B—C1B	1.4 (3)	C7D—C8D—C9D—N2D	0.6 (2)
C7B—N1B—C6B—C1B	−179.8 (2)	C7D—C8D—C9D—C10D	179.9 (2)
N2B—N1B—C6B—C5B	−179.4 (2)	N2D—C9D—C10D—C15D	11.7 (3)
C7B—N1B—C6B—C5B	−0.7 (3)	C8D—C9D—C10D—C15D	−167.5 (2)
N2B—N1B—C7B—C8B	−0.3 (3)	N2D—C9D—C10D—C11D	−168.53 (18)
C6B—N1B—C7B—C8B	−179.1 (2)	C8D—C9D—C10D—C11D	12.2 (3)
N1B—C7B—C8B—C9B	0.1 (3)	C15D—C10D—C11D—C12D	−0.2 (3)
N1B—N2B—C9B—C8B	−0.2 (2)	C9D—C10D—C11D—C12D	−179.91 (18)
N1B—N2B—C9B—C10B	−177.43 (18)	C10D—C11D—C12D—C13D	−0.6 (3)
C7B—C8B—C9B—N2B	0.1 (3)	C10D—C11D—C12D—C16D	177.67 (18)
C7B—C8B—C9B—C10B	177.0 (2)	C11D—C12D—C13D—C14D	0.6 (3)
N2B—C9B—C10B—C15B	5.5 (3)	C16D—C12D—C13D—C14D	−177.66 (18)
C8B—C9B—C10B—C15B	−171.1 (2)	C12D—C13D—C14D—C15D	0.2 (3)
N2B—C9B—C10B—C11B	−177.91 (19)	C13D—C14D—C15D—C10D	−1.0 (3)
C8B—C9B—C10B—C11B	5.4 (3)	C11D—C10D—C15D—C14D	1.0 (3)
C15B—C10B—C11B—C12B	−1.1 (3)	C9D—C10D—C15D—C14D	−179.27 (18)
C9B—C10B—C11B—C12B	−177.69 (19)	Li2—O1D—C16D—O2D	−17.1 (3)
C10B—C11B—C12B—C13B	0.7 (3)	Li2—O1D—C16D—C12D	163.43 (18)
C10B—C11B—C12B—C16B	177.13 (19)	Li1—O2D—C16D—O1D	121.8 (2)
C11B—C12B—C13B—C14B	−0.3 (4)	Li1—O2D—C16D—C12D	−58.7 (3)
C16B—C12B—C13B—C14B	−176.6 (2)	Li1—O2D—C16D—Li2	111.5 (2)
C12B—C13B—C14B—C15B	0.1 (4)	C13D—C12D—C16D—O1D	171.96 (18)
C13B—C14B—C15B—C10B	−0.5 (4)	C11D—C12D—C16D—O1D	−6.3 (3)
C11B—C10B—C15B—C14B	1.0 (4)	C13D—C12D—C16D—O2D	−7.5 (3)
C9B—C10B—C15B—C14B	177.6 (2)	C11D—C12D—C16D—O2D	174.21 (18)
Li2—O1B—C16B—O2B	31.3 (3)	C13D—C12D—C16D—Li2	−165.2 (3)
Li2—O1B—C16B—C12B	−147.2 (2)	C11D—C12D—C16D—Li2	16.6 (4)
C13B—C12B—C16B—O1B	171.3 (2)	O2C—Li2—C16D—O1D	21.7 (2)
C11B—C12B—C16B—O1B	−5.1 (3)	O1B—Li2—C16D—O1D	−109.0 (2)
C13B—C12B—C16B—O2B	−7.4 (3)	O3—Li2—C16D—O1D	147.7 (2)
C11B—C12B—C16B—O2B	176.24 (19)	O2C—Li2—C16D—O2D	−172.6 (2)
O1B—Li2—O2C—C16C	−2.6 (3)	O1B—Li2—C16D—O2D	56.79 (17)
O1D—Li2—O2C—C16C	−120.3 (2)	O1D—Li2—C16D—O2D	165.8 (2)
O3—Li2—O2C—C16C	111.5 (2)	O3—Li2—C16D—O2D	−46.51 (16)
C16D—Li2—O2C—C16C	−128.9 (2)	O2C—Li2—C16D—C12D	−12.3 (5)
C7C—N1C—N2C—C9C	0.3 (2)	O1B—Li2—C16D—C12D	−143.0 (3)
C6C—N1C—N2C—C9C	−178.58 (17)	O1D—Li2—C16D—C12D	−34.0 (4)
C6C—C1C—C2C—C3C	1.2 (3)	O3—Li2—C16D—C12D	113.7 (3)
C1C—C2C—C3C—C4C	−1.5 (4)	O2C—Li2—O3—C17	27.5 (3)
C2C—C3C—C4C—C5C	0.2 (4)	O1B—Li2—O3—C17	148.46 (19)
C3C—C4C—C5C—C6C	1.3 (4)	O1D—Li2—O3—C17	−94.3 (3)
C4C—C5C—C6C—C1C	−1.5 (3)	C16D—Li2—O3—C17	−107.9 (2)
C4C—C5C—C6C—N1C	178.3 (2)	Li2—O3—C17—C18	−133.1 (2)
C2C—C1C—C6C—C5C	0.3 (3)	O1A—Li1—O4—C19	150.1 (2)
C2C—C1C—C6C—N1C	−179.53 (19)	O2D—Li1—O4—C19	27.6 (3)
C7C—N1C—C6C—C5C	−28.2 (3)	O1C^i^—Li1—O4—C19	−91.3 (3)
N2C—N1C—C6C—C5C	150.5 (2)	C16C^i^—Li1—O4—C19	−104.9 (2)
C7C—N1C—C6C—C1C	151.6 (2)	Li1—O4—C19—C20	−133.6 (2)

Symmetry codes: (i) *x*+1, *y*, *z*; (ii) *x*−1, *y*, *z*.

### Hydrogen-bond geometry (Å, °)

**Table:**

*D*—H···*A*	*D*—H	H···*A*	*D*···*A*	*D*—H···*A*
O2A—H1OA···O2C^i^	0.91 (3)	1.70 (3)	2.594 (2)	167 (3)
O2B—H1OB···O2D	0.92 (3)	1.68 (3)	2.584 (2)	165 (3)
O3—H1O3···O1C^i^	0.85 (3)	1.92 (3)	2.761 (2)	171 (3)
O4—H1O4···O1D^i^	0.85 (3)	1.94 (3)	2.780 (2)	170 (2)
C7A—H7AA···N2D	0.93	2.58	3.306 (3)	136
C5C—H5CA···N2B^ii^	0.93	2.60	3.501 (3)	163

Symmetry codes: (i) *x*+1, *y*, *z*; (ii) *x*−1, *y*, *z*.
